# Supplementary material for: Differential Expression and Clinicopathological Significance of HER2, Indoleamine 2,3-Dioxygenase and PD-L1 in Urothelial Carcinoma of the Bladder
Source: J Clin Med. 2020 Apr 27;9(5):1265. doi: 10.3390/jcm9051265 (PMC7288001; doi:10.3390/jcm9051265)
Supplement: Supplementary file 1 [file jcm-09-01265-s001.pdf]

Table S1. Candidate genes associated with CD8+ T cell infiltration in urothelial carcinoma of the bladder.

Rank	Gene Name	Purity-Corrected Partial Spearman's Rho Value	*P* Value
1	PD-L1	0.422	3.03×10^{-17}
2	IDO	0.297	7.27×10^{-9}
3	CTLA4	0.253	9.84×10^{-7}
4	CCL2	0.190	2.46×10^{-3}
5	CCL1	0.153	3.37×10^{-3}
6	CCR2	0.140	7.18×10^{-3}

Table S2. Correlations of HER2, IDO, and PD-L1 expressions with hematologic parameters in 97 patients with urothelial carcinoma of the bladder.

Variable	No.	HER2			IDO			PD-L1 (TCs)			PD-L1 (ICs)		
		Low (SD)	High (SD)	*P*	Low (SD)	High (SD)	*P*	Low (SD)	High (SD)	*P*	Low (SD)	High (SD)	*P*
ANC/μL	97	5748 (2807)	5533 (3732)	0.752 *	6591 (3462)	4808 (2963)	0.007 *	5092 (3029)	6262 (3539)	0.083 *	4944 (2704)	6312 (3716)	0.041 *
ALC/μL	97	1564 (633)	1817 (715)	0.069 *	1536 (638)	1837 (701)	0.030 *	1711 (674)	1681 (707)	0.832 *	1764 (650)	1631 (719)	0.342 *
NLR	97	5.82 (10.29)	4.08 (5.15)	0.287 *	6.72 (10.56)	3.34 (4.40)	0.050 *	3.49 (2.80)	6.53 (11.23)	0.083 *	3.38 (3.03)	6.40 (10.7)	0.063 *

* Unpaired Student's t-test ANC, absolute neutrophil count; ALC, absolute lymphocyte count; NLR, neutrophil to lymphocyte ratio.

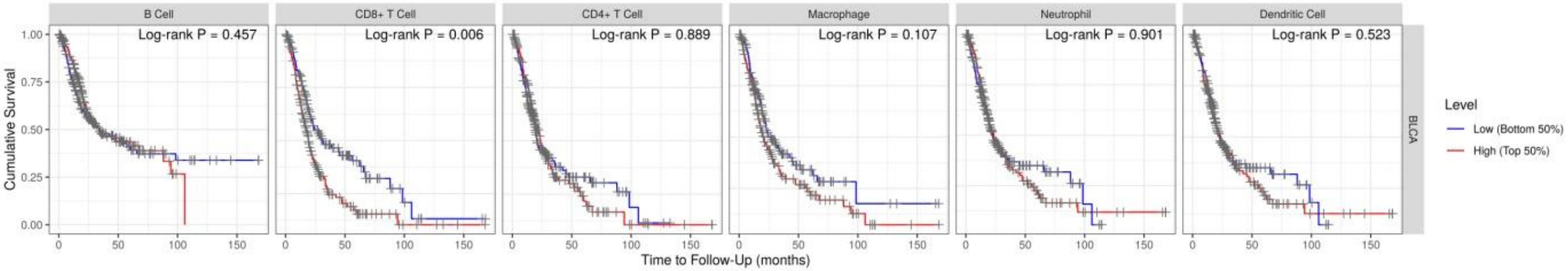

Figure S1. Kaplan-Meier survival curves comparing the high and low infiltrating levels of CD8+ T cells, CD4+ T cells, macrophages, neutrophils, and dendritic cells in urothelial carcinoma of the bladder. Infiltrating levels of CD8+ T cells are significantly correlated with poor OS in urothelial carcinoma of the bladder.
